# Site-specific carbon isotope measurements of vanillin reference materials by nuclear magnetic resonance spectrometry

**DOI:** 10.1007/s00216-022-04292-0

**Published:** 2022-09-13

**Authors:** Phuong Mai Le, Estelle Martineau, Serge Akoka, Gerald Remaud, Michelle M. G. Chartrand, Juris Meija, Zoltán Mester

**Affiliations:** 1 Metrology, National Research Council Canada, 1200 Montreal Road, Ottawa, ON K1A 0R6 Canada; 2 Nantes Université, CNRS, CEISAM, UMR6230, F-44000 Nantes, France; 3 CAPACITÉS SAS, Nantes, France

**Keywords:** Site-specific isotope ratios, Carbon, ^13^C-qNMR, Vanillin, Certified reference material

## Abstract

Vanillin, one of the world’s most popular flavor used in food and pharmaceutical industries, is extracted from vanilla beans or obtained (bio)-synthetically. The price of natural vanillin is considerably higher than that of its synthetic alternative which leads increasingly to counterfeit vanillin. Here, we describe the workflow of combining carbon isotope ratio combustion mass spectrometry with quantitative carbon nuclear magnetic resonance spectrometry (^13^C-qNMR) to obtain carbon isotope measurements traceable to the Vienna Peedee Belemnite (VPDB) with 0.7‰ combined standard uncertainty (or expanded uncertainty of 1.4‰ at 95% confidence level). We perform these measurements on qualified Bruker 400 MHz instruments to certify site-specific carbon isotope delta values in two vanillin materials, VANA-1 and VANB-1, believed to be the first intramolecular isotopic certified reference material (CRMs).

**Graphical abstract:**

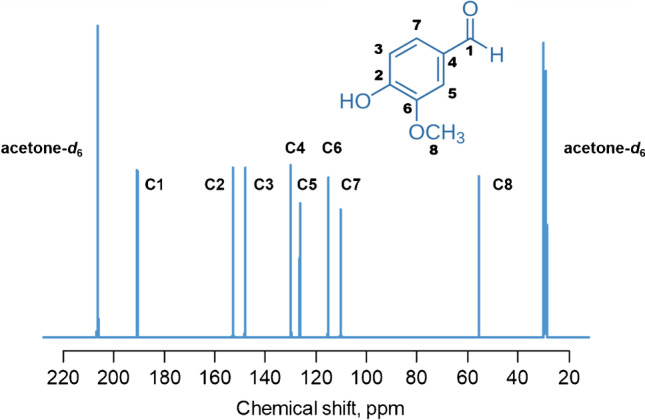

**Supplementary Information:**

The online version contains supplementary material available at 10.1007/s00216-022-04292-0.

## Introduction

**Fig. 1:**
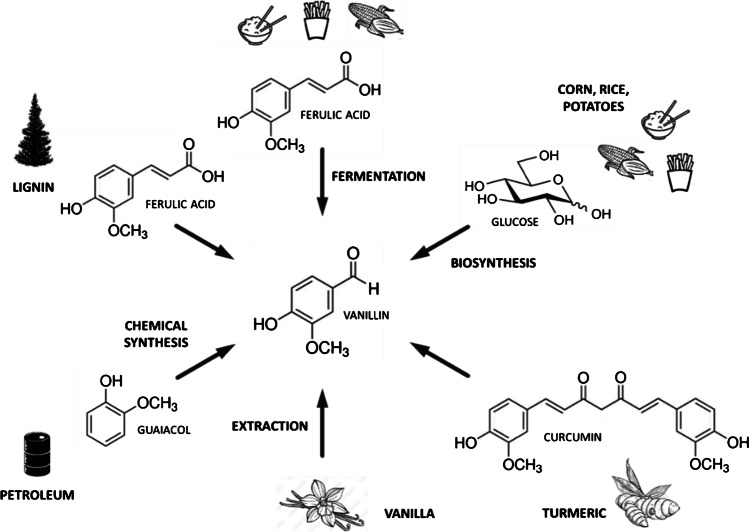
Principal sources of vanillin [5]

Vanillin is one of the most common flavors used in food, cosmetics, and pharmaceuticals. Due to the high cost and demand for vanillin, synthetic vanillin is common. It is estimated that more than 90% of vanillin is from synthetic origins [1], made largely from petrochemicals and by-products from the paper industry (see Fig. 1) [2]. Consumers are increasingly interested in the authenticity and traceability of food products. Chromatographic techniques such as GC and HPLC rely on composition profiles of impurities to distinguish between natural and synthetic vanillin [3] but are unable to detect sophisticated counterfeit methods which add flavoring substances found in vanilla [4].

Stable isotope analysis is valuable for detecting food counterfeiting [6, 7]. Isotope ratio mass spectrometry (IRMS) is commonly used to determine the bulk isotopic composition for a given molecule whereas isotope ratio NMR is capable to determine the site-specific isotopic composition of a given compound.

Isotopic analysis of hydrogen, carbon, and oxygen has been used for the authentication of vanillin products [4, 8–11], with the power of this tool increasing when hydrogen and carbon isotopic compositions are combined [10–12]. Despite this, IRMS provides access to bulk, not to the intramolecular ^13^C composition of the analyte, resulting in some limitations in the authentication of bio- or chemical-synthetic vanillin obtained from the same precursor [9, 11] and detection of the addition of synthetic vanillin to natural vanillin [13]. In contrast, quantitative NMR reveals all spectrally resolved sites in the compound and provides position-specific isotope ratio measurements [14], which enables a more nuanced look into the formation of a molecule by differentiating the sources of the individual carbon atoms [15]. The absolute carbon isotope (^13^C/^12^C) ratio of VPDB references such as glycine reference materials (USGS64, USGS65, and USGS66) has been recently measured using the signals associated with ^12^C and ^13^C (^1^H-^12^C and ^1^H-^13^C signals) in the proton nuclear magnetic resonance spectroscopy (^1^H NMR) [16]. This method provides a new and independent measure of the carbon isotope composition of VPDB, which can calibrate with the IRMS approach to contribute to a better understanding of the isotopic composition of VPDB. However, this method can show some limitations when the overlapping of ^1^H-^12^C and ^1^H-^13^C signals of a given compound are detected. Furthermore, this method can measure the carbon isotope ratio of a given compound, which has a direct connection between carbon and hydrogen, not for quaternary carbons.

The isotopic analysis of vanillin by ^2^H-qNMR proved to be efficient to authenticate natural vanillin extracts using the position-specific isotopic ratio of each protonated position of the molecule [17] and enabled the detection and quantification of additions of up to 10% of synthetic or semi-synthetic vanillin in natural vanillin [13]. However, ^2^H-qNMR requires a long measurement time (5 h acquisition on common spectrometers) and a large sample size (∼1 g of vanillin), and is limited to small molecules because of the complexity of the ^2^H-NMR spectra with a small ^2^H chemical shift range (∼12 ppm at 400 MHz). In addition, hydrogen atoms are easily exchangeable [14]. Furthermore, this method is insufficient to differentiate between the vanillin obtained from ferulic acid by biotechnological processes, and from natural ferulic acid via microorganisms [5].

The analysis of carbon-13 (^13^C-qNMR) presents several advantages over ^2^H, such as improved sensitivity (natural abundance of ∼1.1% and ∼0.015%, for ^13^C and ^2^H, respectively), a larger chemical shift range with fewer overlapping signals, narrower peaks giving a better signal-to-noise ratio, and robustness against chemical exchange [14, 18]. A disadvantage of measuring ^13^C over ^2^H, however, is its much smaller range of variations (isotopic variation in natural compounds ranges from ∼50 to ∼500‰ for ^13^C and ^2^H, respectively, on the δ scale), which requires an NMR method capable of delivering a precision of a few parts per thousand. In order to attain such a high precision, ^13^C-NMR spectra must be recorded with (i) a high signal-to-noise ratio achieved via extensive signal averaging, (ii) nuclear Overhauser effect (nOe) and partial saturation elimination leading to long experimental times, and (iii) an optimized ^13^C-^1^H decoupling sequence in order to perform uniform decoupling over the entire range of ^1^H chemical shifts. These requirements were addressed by using (i) inverse-gated decoupled acquisitions with a 90° flip angle [19], (ii) a large inter-pulse delay [20], and (iii) an adiabatic ^13^C-^1^H decoupling sequence [21, 22]. The site-specific isotopic compositions of carbon are then calculated from the overall (bulk) ^13^C abundance measured by IRMS and from the peak areas observed by ^13^C-qNMR. The trueness and precision of this single-pulse ^13^C-NMR sequence approach has been demonstrated before [19, 20]. In addition, the reproducibility of this method has been addressed through an inter-laboratory collaboration for which vanillin samples were used as the molecular probe on eight similar spectrometers [23].

In this work, we apply the workflow for measuring carbon isotope ratios in vanillin reference materials by NMR for which we provide certified values for each of the constituent carbon atoms.

## Material and methods

### Chemicals

Acetone-*d*_6_, [^13^C_2_]ethanol and [^13^C_2_]acetic acid (both materials containing 0.99 mol/mol enrichment of ^13^C), and dimethyl sulfoxide-*d*_6_ were obtained from Cambridge Isotope Laboratories (Andover, MA, USA). Chromium acetylacetonate (Cr(Acac)_3_, 99%) and HPLC/Spectro grade acetone were obtained from Sigma-Aldrich (Oakville, ON, Canada). IRMS reference materials, IAEA‐CH‐6, USGS65, IAEA-600, NBS22, USGS61, IAEA-603, IAEA-610, IAEA-611, and IAEA-612 were obtained from the International Atomic Energy Agency.

NMR tubes (5 mm inner diameter) were obtained from Wilmad LabGlass (Buena, NJ, USA). Two high-purity synthetic vanillin materials were obtained from NRC [24]. Maleic acid (SRM grade, TraceCERT®, having a purity of 0.9989 ± 0.0031 g/g) was obtained from Fluka. VANA-1 and VANB-1 are both synthetic high-purity vanillin materials obtained from Fisher Scientific, Waltham, MA, USA) and from Sigma-Aldrich (St. Louis, MO, USA), respectively.

### IRMS analyses

The bulk isotopic composition of carbon in VANA-1 and VANB-1 was determined by elemental analysis combustion IRMS. Specifically, the carbon isotope delta value relative to the VPDB, *δ*(^13^C), was determined at NRC. Details of the standard procedure for *δ*(^13^C) measurements are described by Chartrand et al. [24]. Briefly, approx. 650 µg of vanillin samples and an appropriate amount of carbon isotope reference materials were weighed into 5 × 3.5 mm tin capsules (Elemental Microanalysis; Okehampton, UK) and were loaded onto an elemental analyzer (Vario EL III; Elementar Americas Inc., Mt. Laurel, NJ, USA) interfaced with a gas flow controller (Conflow III; Thermo Fisher; Bremen, Germany) to an isotope ratio mass spectrometer (Delta^+^XP; Thermo Fisher; Bremen, Germany). Combustion and reduction reactors were set to 950 °C and 500 °C, respectively. Helium dilution on the Conflow III was set to 0.5 bar (7 psi) pressure. Carbon isotope delta measurements were calibrated on the VPDB scale using nine reference materials: IAEA‐CH‐6, USGS65, IAEA-600, NBS22, USGS61, IAEA-603, IAEA-610, IAEA-611, and IAEA-612. The carbon isotope delta values for VANA-1 and VANB-1 relative to the VPDB have been determined to be − 31.30 ± 0.06‰ and − 25.85 ± 0.05‰ (expanded uncertainties are at 95% confidence), respectively. Details regarding the assignment of these values are described by Chartrand et al. [25].

### NMR analyses

#### ^**1**^**H-qNMR**

The chemical purity of vanillin in VANA-1 and VANB-1 was determined by quantitative ^1^H-NMR on a Bruker-NMR 400 spectrometer (Bruker Optics Inc; MA, USA) operating at 400.13 MHz for proton (^1^H) resonance frequency equipped with a 5 mm ID NMR probe controlled at 298 K, following a method adapted from Le et al. [26]. In brief, three replicate samples of each vanillin sample (15 mg) and maleic acid (8 mg) as an internal calibrator were weighed on an analytical microbalance (UMT-2; Mettler-Toledo) and co-dissolved in 2 mL DMSO-*d*_6_ in a 20-mL vial. The solutions were thoroughly vortex-mixed and 0.8 mL of each sample solution was transferred into an NMR tube. The ^1^H-qNMR acquisition was performed with a 90° pulse, a sweep width of 15 ppm (6000 Hz), 32 scans of 32,000 data points preceded by 2 dummy scans, an acquisition time of 2.72 s, and a recovery delay of 45 s (> 7*T*_1,max_) to ensure accuracy of 0.1% with a 90° pulse. Data processing was performed using 128,000 data points. The FIDs were Fourier transformed and apodized by an exponential function leading to a line broadening of 0.3 Hz (LB). The ^1^H-qNMR spectra were manually phased and a third-order polynomial baseline correction was applied manually. The signals used for quantitation of vanillin correspond to the following regions of the spectra: 7.70–7.10 ppm (2H, H_7_ and H_5_), 7.07–6.70 ppm (1H, H_3_), 4.10–3.60 ppm (3H, OCH_3_), and 6.56–5.98 ppm (2H, maleic acid) as shown in Figure S1 (see Electronic Supplementary Material). The reference frequency was set to the DMSO-*d*_6_ signal at 2.50 ppm. All spectra were integrated using TopSpin software (v3.6, Bruker) and included the ^13^C satellite signals.

#### ^**13**^**C-qNMR**

Quantitative ^13^C-NMR spectra were recorded at 100.62 MHz using two Bruker Avance III 400 spectrometers—one in Ottawa (Canada) and the other in Nantes (France)—both equipped with a 5 mm i.d. NMR-BBFO probe. The temperature of the probe was set to 303 K. Probe tuning and matching were performed at the recording frequency of 100.62 MHz. Both ^13^C and ^1^H channels were automatically tuned and matched, followed by manual verification to check the obtained results.

##### Spectrometer adjustments and qualification

**Fig. 2:**
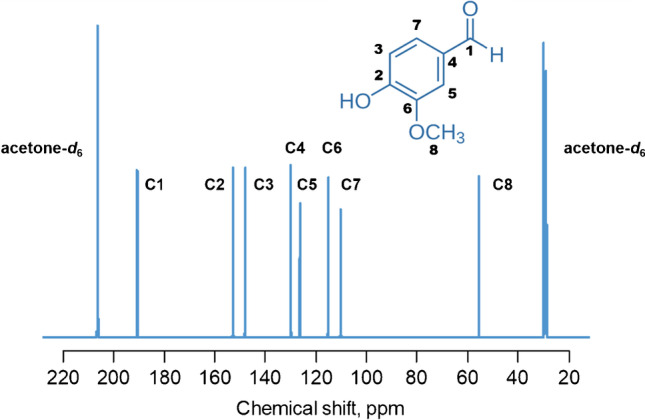
^13^C-qNMR spectrum of vanillin in acetone-*d*_6_ recorded at NRC on 400 MHz NMR instrument. Numbering of carbon atoms is in the order of decreasing ^13^C chemical shift

The carbon site-specific isotope delta values of the vanillin molecule, *δ*(^13^C_*k*_) where *k* = 1…8 (see Fig. 2 for carbon numbering), were determined using the single-pulse ^13^C-NMR sequence mentioned above. Details of the systematic protocol for NMR spectrometer and experimental setup are described by Chaintreau et al. [23].

Determination of the decoupling power attenuation was performed as follows. A series of ^13^C spectra of a 10% CHCl_3_ sample in acetone-*d*_6_ were acquired using the decp90 pulse program. The transmitter excitation frequencies O1p (for ^13^C) and O2p (for ^1^H) were set to *δ*(^13^C) = *δ*(^13^C,CHCl_3_) + 0.025 ppm and *δ*(^1^H) = *δ*(^1^H,CHCl_3_) + 0.1 ppm. The first ^13^C-qNMR spectrum was recorded with an impulsion of 14.1 µs (p3) with the corresponding power level (parameter PL12), delay of 20 s equal to *J*/2 (with *J* = 212 Hz), and ^1^H power level (PL2 = 120 dB). The experiment was repeated by decreasing PL2 until the ^13^C doublet signal disappears, which corresponds to the 90° ^1^H pulse (PL2dec = 13 W). This decoupling attenuation value was used as the decoupling power in the inverse-gated sequence for the adiabatic decoupling.

NMR spectrometer qualification was performed as follows. The adiabatic decoupling pulse and CPD program for adiabatic decoupling were inserted into the ^13^C-NMR (zgig) existing pulse sequence in the NMR spectrometer. The ability of the NMR for the proton decoupling conditions was evaluated by using [^13^C_2_] ethanol. When adiabatic decoupling was used, the acquisition time was limited to 1 s to control the heating effect of decoupling. The ^13^C NMR spectrum of the sealed tube was recorded under quantitative conditions of inverse-gated ^1^H decoupling and repetition time *d*_1_ = 20 s (> 10*T*_1,max_) to avoid partial saturation and build-up of the nOe. Thirteen acquisitions of ^13^C with ^1^H-decoupling were performed with a systematic variation of the ^1^H decoupling offset set to *ν*_0_ = ½*ν*(^1^H,CH_3_) + ½*ν*(^1^H,CH_2_) for ethanol, ranging between *ν*_0_ ± 3000 Hz with 500 Hz increments with five spectra recorded for each offset value.

Doubly-labelled [^13^C_2_] ethanol was used to evaluate the NMR performance as described in detail below. In short, stoichiometry dictates that the peak areas of the two doublets are identical and this was used to evaluate the accuracy of the isotope ratio measurements by ^13^C-qNMR [23].

##### Measurements of vanillin samples

Approximately 250 mg of vanillin was dissolved in 400 µL of acetone-*d*_6_ followed by the addition of 100 µL of 0.1 M Cr(Acac)_3_ (in acetone) relaxation solution using a Hamilton glass syringe. The resulting solution was vortex-mixed and then equilibrated at room temperature for approx. 1 h. The vanillin solution was carefully filtered to remove any non-dissolved particles of Cr(Acac)_3_ before transferring it to the NMR tube. The NMR tube was sealed with a plug and parafilm to prevent evaporation of the solvent.

The temperature of the probe and sample was set at 303 K. The offsets for both ^13^C and ^1^H were set at the middle of the frequency range: O1p at 119 ppm (for ^13^C) and O2p at 6.5 ppm (for ^1^H). Inverse-gated decoupling was applied to avoid the nOe. The longitudinal relaxation time (*T*_1_) of ^13^C nuclei was determined by using an inversion recovery sequence, with 8 inversion-time values ranging from *τ* = 0.1 s to 20 s, and by using the *T*_1_ processing software of the spectrometer. For most vanillin samples, the 90° ^13^C pulse widths were calibrated to 9.9 µs. All *T*_1_ values of ^13^C nuclei were less than 1.6 s. Thus, the repetition delay between each 90° pulse was fixed at 20 s (> 10*T*_1,max_) to achieve quantitative relaxation of the magnetization and nOe cancellation. The decoupling sequence used adiabatic full-passage pulses with cosine square amplitude modulation (*ν*_2,max_ = 17.6 kHz) and offset independent adiabaticity with optimized frequency sweep as described elsewhere [21]. The acquisition conditions were as follows: acquisition time of 0.7 s, repetition delays of 20 s, 4 dummy scans, and 400 sample scans, resulting in the overall acquisition time of 140 min per spectrum. Five spectra were consecutively acquired for each sample. A typical ^13^C-qNMR spectrum of vanillin is shown in Fig. 2.

Samples of VANA-1 and VANB-1 were measured in Ottawa and additional samples of VANA-1 and VANB-1 were also measured in Nantes with the same ^13^C-qNMR parameters except for the ^13^C-pw90 for where the value optimized for vanillin on the Nantes instrument was used.

#### **NMR data processing**

Data were processed using the TopSpin software (v3.6) with the same parameters and the same procedure for free induction decays of 256 K: (i) an exponential multiplication inducing a 2 Hz line broadening, (ii) manual phasing, and (iii) automatic baseline correction using a third-order polynomial function. The carbon signal peak areas were calculated in accordance with a frequency domain mathematical model using Rnmrfit package in R [27] using the following integration windows: 191.7–189.7 ppm (C1); 153.7–151.7 ppm (C2); 149.0–147.0 ppm (C3); 130.8–128.8 ppm (C4); 127.4–125.4 ppm (C5); 116.2–114.2 ppm (C6); 111.2–109.2 ppm (C7); and 56.6–54.4 ppm (C8).

#### Site-specific carbon isotope delta values

The carbon isotope specific-position for each vanillin was established and traced based on the bulk carbon isotopic composition obtained by IRMS. For subsequent model calculations, we have adopted the ^13^C/^12^C isotope ratio *R*_VPDB_ = 0.011108 for the VPDB which is the weighted average of three recent independent estimates [16, 28, 29].

Carbon isotope ratios for each carbon atom were calculated from the processed spectra using the measurement model of Silvestre et al. [30] as outlined below. The input data are the areas corresponding to the main (central) peaks observed in ^13^C-NMR spectra. The established procedure is to adjust these measured peak areas (*S*) to account for the slight loss of intensity caused by the satellite peaks arising from the ^13^C–^13^C interactions [23]:

1 $${S}_{i,\mathrm{adj}}={S}_{i}\left(1+0.011{n}_{i}\right)$$

where *n*_*i*_ is the number of carbon atoms directly bonded to the carbon atom corresponding to each NMR signal. For example, C1 (*i* = 1) is directly bound to C4 therefore *n*_1_ = 1 whereas C4 is directly bound to C1, C5, and C7 therefore *n*_4_ = 3.

The isotopic distribution of vanillin is first characterized by the relative share of ^13^C for each carbon atom:

2 $${f}_{i}{(}^{13}\mathrm{C})={S}_{i,\mathrm{adj}}/\left({S}_{1,\mathrm{adj}}+{S}_{2,\mathrm{adj}}+\dots +{S}_{8,\mathrm{adj}}\right)$$

The relative share of ^13^C when all eight atoms have identical isotopic composition is:

3 $${F}_{i}{(}^{13}C)={P}_{i}/\left({P}_{1}+{P}_{2}+\dots +{P}_{8}\right)$$

where *P*_*i*_ is the number of equivalent carbon atoms for the molecular position *k*. Each peak observed in the ^13^C-NMR spectrum of vanillin presents a single equivalent carbon (Fig. 2); therefore *P*_*i*_ = 1 for *i* = 1…8 and *F*_*i*_ = 1/8.

The average abundance of ^13^C in the vanillin sample, *x*(^13^C), is obtained from *δ*(^13^C) measurements by IRMS and the ^13^C/^12^C reference value for VPDB:

4 $${R}_{13}={R}_{\mathrm{VPDB}}\left[1+{\delta (}^{13}\mathrm{C})\right]$$

5 $${x(}^{13}\mathrm{C})={R}_{13}/\left(1+{R}_{13}\right)$$

This average value is used to obtain the isotopic abundance of carbon-13 at each position *k*:

6 $$x_i(^{13}\mathrm C)={x(}^{13}\mathrm C)\cdot f_i/F_i$$

Similarly, the ^13^C/^12^C isotope ratio is calculated for each carbon atom:

7 $${R}_{13,i}={x}_{i}{(}^{13}\mathrm{C})/\left[1-{{x}_{i}(}^{13}\mathrm{C})\right]$$

Lastly, one obtains carbon isotope delta values for each carbon atom in vanillin:

8 $${\delta }_{i}{(}^{13}\mathrm{C})={R}_{13,i}/{R}_{\mathrm{VPDB}}-1$$

**Fig. 3:**
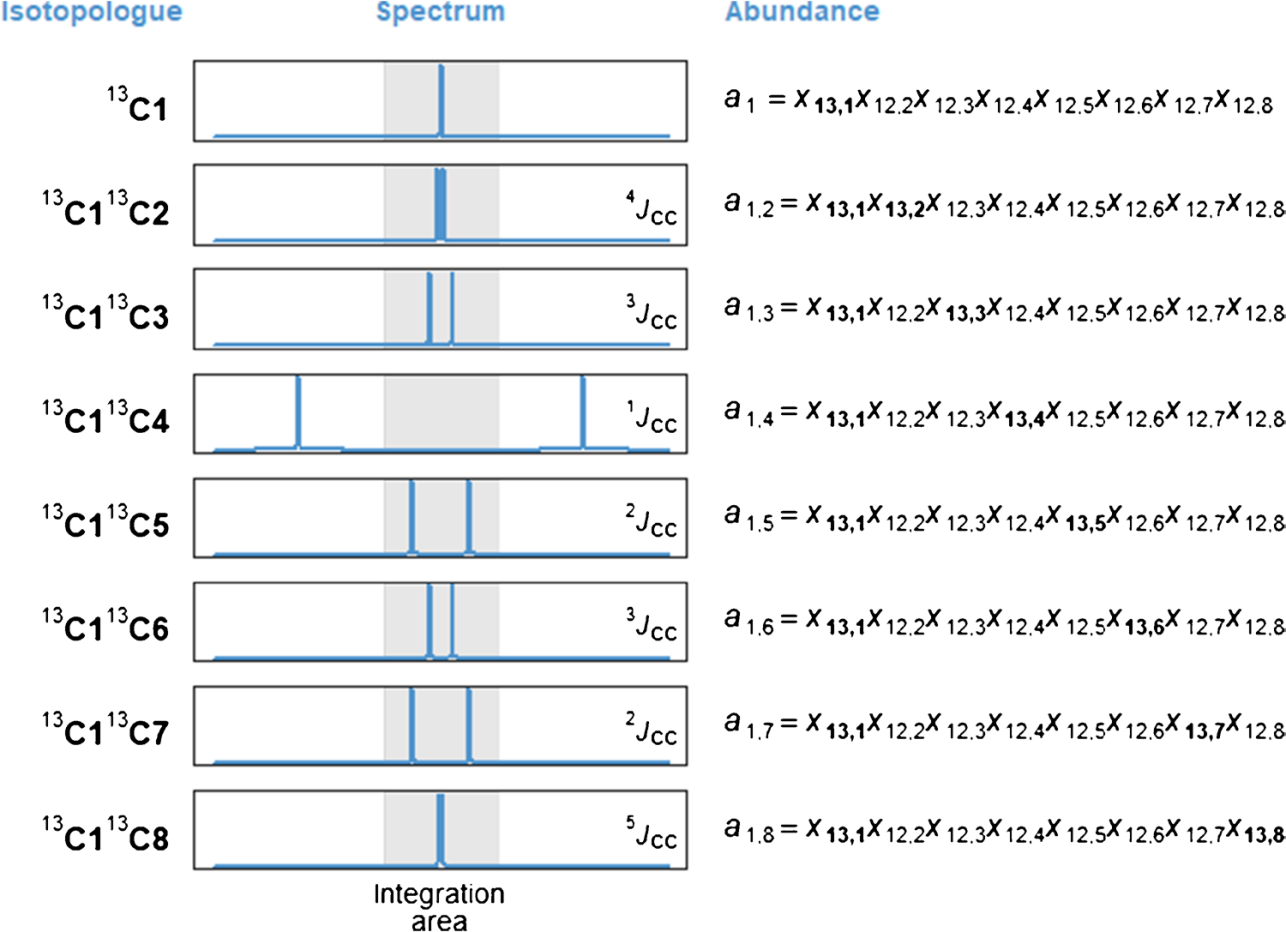
Schematic components of the C1 carbon atom peak in the ^13^C-qNMR spectrum of vanillin. Note that only the ^13^C–^13^C interactions are observed in this acquisition mode. The observed peak area for C1 is proportional to the sum of these components with the exception of ^13^C1^13^C4 isotopologue (*a*_1,4_) which is outside the integration area of the main peak

In order to further investigate the above measurement model, we note that Eqs. 1–8 (which we term model M0) assume an identical abundance of ^13^C (1.1%) for all carbon atoms in the clumped isotopologues having two or more C_13_ atoms, instead of using the actual abundances to perform the correction of signal areas. To overcome this simplification, one can identify the specific isotopologues that make up the central (main) peaks integrated into this study. Figure 3 shows such a schematic for the C1 signal. Taken these considerations together, one can establish a more refined measurement model for vanillin in Eqs. 9–16.

The measurement model for site-specific carbon isotope delta values in vanillin can be written in the form of a generative model which lends itself more suitable for Bayesian analysis (see Supplementary file), with the observed peak areas for vanillin carbon atoms, *S*_*i*_ (*i* = 1…8), modelled as an outcome of (unknown) NMR sensitivity factor, *s*, and (unknown) isotopic abundances of carbon-13 at each site, *x*_13,*i*_:

9 $${S}_{1}=s\left({a}_{1}+{a}_{1,S}-{a}_{\mathrm{1,4}}\right)$$

10 $${S}_{2}=s\left({a}_{2}+{a}_{2,S}-{a}_{\mathrm{2,3}}-{a}_{\mathrm{2,6}}\right)$$

11 $${S}_{3}=s\left({a}_{3}+{a}_{3,S}-{a}_{\mathrm{3,2}}-{a}_{\mathrm{3,7}}\right)$$

12 $${S}_{4}=s\left({a}_{4}+{a}_{4,S}-{a}_{\mathrm{4,1}}-{a}_{\mathrm{4,5}}-{a}_{\mathrm{4,7}}\right)$$

13 $${S}_{5}=s\left({a}_{5}+{a}_{5,S}-{a}_{\mathrm{5,4}}-{a}_{\mathrm{5,6}}\right)$$

14 $${S}_{6}=s\left({a}_{6}+{a}_{6,S}-{a}_{\mathrm{6,2}}-{a}_{\mathrm{6,5}}\right)$$

15 $${S}_{7}=s\left({a}_{7}+{a}_{7,S}-{a}_{\mathrm{7,3}}-{a}_{\mathrm{7,4}}\right)$$

16 $${S}_{8}=s\left({a}_{8}+{a}_{8,S}\right)$$

Parameters *a*_*i*_ and *a*_*i*,*j*_ (*i*, *j* = 1…8) are as described in Fig. 3 and *a*_*i*,S_ = sum{*a*_*i*,*k*_} (*k* = 1…8). These equations are solved for *s* and *x*_13,*i*_ (*i* = 1…8) using numerical methods along with the constraint imposed on the values of *x*_13,*i*_ that the individual isotope values, *δ*_*i*_(^13^C), calculated using Eqs. 7–8, must average to the value provided by IRMS. In the simplest interpretation, Eqs. 9–16 are parametrized in terms of eight unknown ^13^C abundances, *x*_13,*i*_ (*i* = 1…8). This assumes, for example, that the isotopic abundance of ^13^C in the C1 atom is the same in all isotopologues containing the C1 carbon atom shown in Fig. 3. We term this model M1. This nonlinear measurement model can be solved using standard numerical solver procedures available in either Excel (see Electronic Supplementary Material) or with dedicated mathematical software such as Mathematica or R. Our results show that the sensitivity factor (*s*) varies by approx. 1–3 parts per thousand between the replicate measurements of vanillin samples. It is simply a scaling factor between the (relative) signals predicted by the model and the observed peak areas.

In reality, there might be some variation in the isotopic abundances of ^13^C (in the carbon atoms of the same position in the vanillin molecule) among the various isotopologues. This variability can be addressed using hierarchical random effects models, similar to how between-unit homogeneity is commonly evaluated in the reference materials. This, however, is outside the scope of this work.

## Results and discussion

### ^1^H-qNMR for purity determination

Determination of the site-specific carbon isotope profile by ^13^C-qNMR requires high-purity (> 99%) organic materials [31]. In many cases, this can be achieved by the use of commercial chemicals. We used the ^1^H-qNMR technique with maleic acid qNMR internal standard to evaluate the chemical purity of vanillin in both VANA-1 and VANB-1 [32]. The integration regions in the ^1^H-qNMR measurements are wide enough to contain the adjacent ^13^C satellites and the integrated signal area: for vanillin material 7.70–7.10 ppm: 2H (H_5_ and H_7_); 7.07–6.70 ppm: 1H (H_3_); and 4.10–3.60: 3H–OCH_3_; for maleic acid as IS 3H: 6.56–5.98 ppm (as described in “^1^H-qNMR”).

The purity of vanillin in VANA-1 and VANB-1 materials of 0.9982 g/g and 0.9973 g/g, respectively determined using ^1^H-qNMR, is thus suitable to use for the determination of the carbon isotope profile of these materials. The assignment of these values is described in Electronic Supplementary Material Table S1.

### ^13^C-NMR pulse sequence for carbon isotope site-specific measurement

**Fig. 4:**
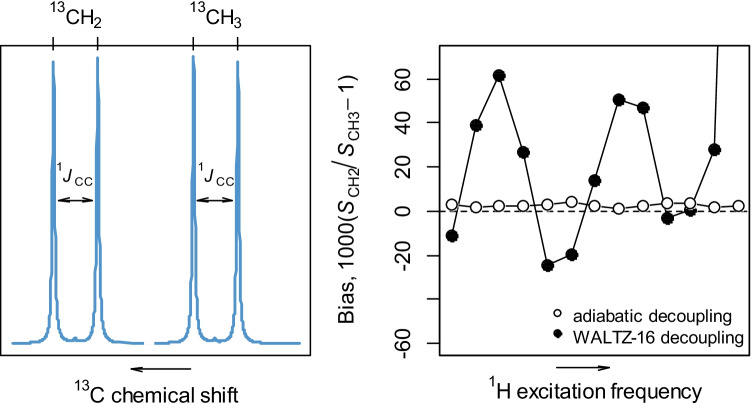
NRC 400 MHz NMR qualification using adiabatic and standard WALTZ-16 ^13^C-^1^H decoupling pulses on the doubly-labelled [^13^C_2_]-ethanol

The main difficulty with obtaining high-precision isotope ratio values with ^13^C-NMR lies with ensuring uniform ^1^H decoupling efficiency over the whole range of ^1^H chemical shifts. This problem has been solved with the help of an adiabatic ^1^H decoupling sequence using a numerical calculation of the frequency sweep from an amplitude evolution with a cosine shape [21]. The performance qualification of the NMR spectrometer was evaluated using doubly-labelled [^13^C_2_]ethanol as a molecular probe to demonstrate uniform and homogeneous ^1^H decoupling efficiency over a large range of resonances [23]. The ^13^C-qNMR spectrum of [^13^C_2_] ethanol shows four main peaks (Fig. 4)—a doublet for ^13^CH_2_ and a doublet for ^13^CH_3_ resonances. The expected area of these two doublets is *S*_CH2_/*S*_CH3_ = 1, irrespective of the precise isotopic composition of either of the carbon atoms, and this was used to evaluate the accuracy of ^13^C-qNMR. Any deviation from this expected ratio would indicate a lack of accuracy in the NMR configuration used and for our purposes this deviation should be within few parts per thousand (1–2‰) [23].

When applied to the 400 MHz NMR at NRC, this instrument qualification procedure showed a deviation of ≤ 2‰ over the wide range of ^1^H excitation frequencies (Fig. 4), thus indicating that our NMR spectrometer is suitably qualified to measure ^13^C/^12^C isotope ratios at natural abundances. We also compared the adiabatic ^13^C-^1^H-decoupling scheme to the WALTZ-16 standard pulse on doubly-labelled ethanol (Fig. 4). The latter pulse sequence shows significant biases indicating that the WALTZ-16 ^13^C-^1^H-decoupling is sensitive to off-resonance effects and to the presence of ^1^H-^1^H couplings and is not suitable for high-precision carbon isotope ratio measurements by NMR [21]. To obtain an estimate of how reliable the peak area measurements are for vanillin, a similar qualification procedure was performed using doubly-labelled [^13^C_2_]-acetic acid. The average deviation from the 1:1 ratio of the two doublets (–COOH and –CH_3_) is approx. 2‰, identical to the ethanol.

### Isotopic composition of vanillin by ^13^C-qNMR

#### **Isotopic**.^**13**^**C NMR experiments applied on VANA-1 and VANB-1**

Isotope ratio precision of ^13^C-qNMR measurements depends mainly on the signal-to-noise ratio achievable in a reasonable period of time which, in turn, is a function of the acquisition time, the delay between pulses, and the number of scans [14]. The vanillin molecule presents several challenging technical features: a wide range of chemical shifts for carbon atoms (135 ppm) and hydrogen atoms (6 ppm), long relaxation times (up to 19 s), and a wide range of nOe factors. These issues were resolved with the help of an optimized adiabatic ^1^H decoupling sequence [19, 21] and 0.1 M Cr(Acac)_3_ paramagnetic relaxation solution. At this concentration, Cr(Acac)_3_ does not affect the sensitivity and accuracy of ^13^C-qNMR [20], while a large reduction of the *T*_1_ values (from 19 to 1.6 s) for each carbon in vanillin is observed upon the addition of Cr(Acac)_3_. This provides a significant reduction of the inter-pulse delay thus allowing a tenfold reduction of the overall acquisition time [18, 20]. In our study, the longest *T*_1_ of 1.57 s was observed at C4 carbon in vanillin (Table 1). In such conditions, the achieved peak area precision of 1–2 parts per thousand was achieved for the determination of the position-specific isotopic analysis of VANA-1 and VANB-1. Setting the acquisition time up to 0.7 s for adiabatic decoupling ensured probe integrity as well as avoided the heating effect of decoupling and nOe. The use of 400 scans for 250 mg of vanillin solution in 500 µL acetone-*d*_6_ with 0.1 M Cr(Acac)_3_ (in acetone) solution resulted in the signal-to-noise ratio S:N > 720 for all carbon peaks in the vanillin samples.

**Table 1:** Longitudinal relaxation times of each carbon in vanillin in the presence of 0.1 M Cr(Acac)_3_^a^

	C1	C2	C3	C4	C5	C6	C7	C8
*T*_1_/s	1.03 (0.07)	1.26 (0.09)	1.46 (0.08)	1.57 (0.05)	0.85 (0.04)	0.84 (0.05)	0.93 (0.03)	0.86 (0.06)

^a^Values in the parenthesis are the standard deviations of *N* = 20 replicate measurements

#### **Site-specific **^**13**^**C content in vanillin samples**

Using the optimized experimental conditions described above, we have performed site-specific carbon isotope measurements in reference materials VANA-1 and VANB-1. First, we discuss the effect of the measurement model. We note that the measurements of a single vanillin sample can provide carbon isotope delta values that differ by up to 1‰ between the two measurement models outlined in this work (Table 2). In this work, we adopt the arithmetic average of these two models.

**Table 2:** The effect of measurement model on the carbon isotope delta results for a single measurement of vanillin in VANA-1 sample from vial number 170 at NRC

Model	C1	C2	C3	C4	C5	C6	C7	C8
M0	− 20.8	− 30.7	− 33.4	− 28.7	− 28.0	− 30.0	− 24.0	− 54.8
M1	− 20.4	− 31.0	− 33.6	− 29.2	− 28.2	− 30.2	− 24.3	− 53.6

The site-specific carbon isotope delta values, *δ*_*i*_(^13^C), measured at the NRC by ^13^C-qNMR for VANA-1 and VANB-1 samples, are presented in Tables 3–4. The *δ*_*i*_(^13^C) values are expressed in permilles relative to the VPDB, five measurements for each bottle. The consistency of *δ*_*i*_(^13^C) values from sample to sample range from 0.3 to 1.1‰ for both VANA-1 and VANB-1, as evaluated by the repeatability standard deviations from NRC measurements shown in Tables 3–4.

**Table 3:** Site-specific average carbon isotope delta values of vanillin in VANA-1 samples measured at NRC

VANA-1 vial number	C1	C2	C3	C4	C5	C6	C7	C8
120	− 22.1	− 31.8	− 32.6	− 28.4	− 29.6	− 29.9	− 23.2	− 52.8
170	− 21.1	− 32.1	− 33.1	− 29.2	− 29.4	− 29.3	− 22.8	− 53.5
290	− 21.1	− 32.6	− 31.8	− 27.9	− 30.6	− 30.0	− 23.0	− 53.4
380	− 21.8	− 32.4	− 31.4	− 27.0	− 30.4	− 29.8	− 24.2	− 53.3
380	− 20.9	− 31.9	− 32.7	− 27.7	− 30.4	− 28.6	− 25.1	− 53.1
510	− 21.5	− 31.8	− 34.0	− 28.8	− 28.9	− 27.9	− 24.4	− 53.2
540	− 21.2	− 31.5	− 32.8	− 28.6	− 29.4	− 30.0	− 23.4	− 53.6
650	− 22.0	− 32.3	− 32.5	− 27.7	− 29.8	− 29.2	− 23.6	− 53.4
870	− 20.8	− 33.3	− 32.6	− 28.7	− 29.8	− 29.6	− 22.2	− 53.5
910	− 21.2	− 31.3	− 31.7	− 28.6	− 30.1	− 29.5	− 25.5	− 52.7
**Mean**	− **21.4**	− **32.1**	− **32.5**	** − 28.3**	** − 29.8**	** − 29.4**	** − 23.7**	** − 53.2**
SD	0.5	0.6	0.8	0.7	0.6	0.7	1.0	0.3

The values in bold are mean values. Standard deviations are given one row below

**Table 4:** Site-specific average carbon isotope delta values of vanillin in VANB-1 samples measured at NRC

VANB-1 vial number	C1	C2	C3	C4	C5	C6	C7	C8
5	− 19.3	− 31.2	− 33.6	− 24.6	− 22.1	− 26.9	− 18.6	− 30.7
115	− 19.9	− 29.7	− 32.6	− 24.8	− 24.8	− 26.5	− 18.6	− 29.9
465	− 20.8	− 29.9	− 33.1	− 24.1	− 24.8	− 26.7	− 17.2	− 30.2
585	− 20.9	− 29.6	− 31.9	− 23.9	− 25.3	− 26.5	− 18.4	− 30.4
715	− 20.5	− 29.9	− 33.2	− 24.5	− 23.5	− 26.9	− 18.1	− 30.2
725	− 20.4	− 30.5	− 33.0	− 23.5	− 24.8	− 26.3	− 18.4	− 30.0
785	− 19.8	− 29.5	− 32.2	− 25.6	− 23.3	− 24.9	− 19.9	− 31.5
855	− 19.6	− 29.6	− 33.0	− 24.6	− 22.7	− 26.0	− 20.5	− 30.9
905	− 20.3	− 29.6	− 32.1	− 24.3	− 24.4	− 27.2	− 17.9	− 31.0
995	− 19.3	− 29.4	− 33.0	− 24.9	− 22.9	− 25.7	− 20.6	− 31.1
**Mean**	** − 20.1**	** − 29.9**	** − 32.8**	** − 24.5**	** − 23.9**	** − 26.4**	** − 18.8**	** − 30.6**
SD	0.6	0.5	0.5	0.6	1.1	0.7	1.1	0.5

The values in bold are mean values. Standard deviations are given one row below

VANA-1 and VANB-1, have average *δ*(^13^C) values of − 31‰ and − 26‰ relative to the VPDB, respectively, consistent with synthetic origin. Vanillin from petrochemical origin typically has *δ*(^13^C) values from − 36 to − 25‰ and lignin-based biovanillin has *δ*(^13^C) values from − 29 to − 26‰ [2, 5]. Thus, these can be distinguished from natural vanillin from the vanilla pod having *δ*(^13^C) values from − 22 to − 14‰ [11]. Although the average isotopic composition of vanillin from VANA-1 and VANB-1 are somewhat similar, the difference in the isotopic composition of carbon C8 (OCH_3_) between these two materials is extremely large, − 54‰ and − 31‰, which likely reflects the different synthetic routes for these vanillin samples. In addition, we observe substantial variations in the isotopic composition of carbon atoms in the benzene ring of vanillin for both VANA-1 and VANB-1. Both materials exhibit approximately 10‰ difference between the individual carbon atoms—well in excess of the repeatability standard deviation (< 1‰). In both materials, C7 carbon atoms show the highest abundance of ^13^C and the C2 atoms the lowest.

### Inter-laboratory comparison

To support the NRC certification work, an inter-laboratory comparison study was conducted with Nantes laboratory (France), which also used an adiabatic pulse sequence for ^1^H decoupling on the 400 MHz spectrometer. To avoid variability associated with the NMR data processing, the free induction decays from vanillin samples were processed by the NRC using the same software and analysts. Given that NRC measurements were obtained in two separate measurement campaigns, we consider our results as if they are from three laboratories. The median standard deviation between the laboratory results is 1‰ (Tables 5–6).

**Table 5:** Inter-laboratory measurements of carbon isotope deltas in VANA-1

Laboratory	C1	C2	C3	C4	C5	C6	C7	C8
NRC (Apr 2021)	− 21.3	− 32.3	− 32.6	− 28.6	− 29.8	− 29.8	− 22.9	− 53.4
NRC (Jun–Oct 2021)	− 21.5	− 31.9	− 32.5	− 28.0	− 29.9	− 29.0	− 24.5	− 53.1
Nantes (Feb 2020)	− 22.5	− 31.7	− 32.6	− 31.0	− 29.6	− 26.7	− 25.6	− 50.7
SD	0.7	0.3	0.1	1.6	0.2	1.6	1.3	1.5

**Table 6:** Inter-laboratory measurements of carbon isotope deltas in VANB-1

Laboratory	C1	C2	C3	C4	C5	C6	C7	C8
NRC (Mar–Apr 2021)	− 20.0	− 30.0	− 32.7	− 24.7	− 23.9	− 26.5	− 18.4	− 30.7
NRC (Jun–Oct 2021)	− 20.1	− 29.8	− 32.8	− 24.3	− 23.8	− 26.3	− 19.2	− 30.5
Nantes (Feb 2020)	− 21.7	− 31.4	− 34.0	− 25.9	− 21.5	− 24.4	− 20.3	− 27.7
SD	0.9	0.9	0.7	0.9	1.4	1.2	1.0	1.7

### Isotope measurements on high-field NMR spectrometer

Tables 5–6 indicate that close agreement between the results of site-specific isotope values can be obtained with two qualified Bruker 400 MHz spectrometers in Nantes and Ottawa. In this work, we also compare the results obtained from a Bruker 400 and Bruker Avance HD 700 MHz spectrometers. The uniform decoupling over the entire range of ^1^H chemical shifts of the doubly-labelled ethanol on the 400 and 700 MHz spectrometers is 2‰ and 5‰, respectively. This difference can be explained by differences in the coil architecture: a BBFO probe on the 400 MHz and a cryogenically cooled probe on the 700 MHz. Differences in the shape and the length of each coil (for ^1^H and ^13^C) may result in ^1^H decoupling deficiency (^1^H coil) or edge effects (^13^C coil). In the case of vanillin, we observed that the values of carbon isotopic composition of VANA-1 and VANB-1 on 700 MHz spectrometers are significantly different from those measured on the 400 MHz (Table 7): the value for C8 in VANA-1 observed from 700 MHz instrument is − 78‰ whereas the average value from 400 MHz instrument is − 53‰. However, the relative difference between the measured values of VANA-1 and VANB-1 remains nearly the same: 23‰ (700 MHz) and 24‰ (400 MHz). This finding further indicates that reliable isotope delta measurements require careful qualification and calibration of the instrument. Additionally, the measurements on 700 MHz instrument can be calibrated against the VANA-1 with the correction factors for each carbon atom (see Electronic Supplementary Material Table S2). The current single-pulse ^13^C-NMR sequence needs to be further investigated for incorporation with the high-field NMR such as 700 MHz. Currently, however, the high-field NMR is not particularly well-suited for these measurements.

**Table 7:** Measurements of VANA-1 and VANB-1 on 700 MHz NMR instrument

Atom	*δ*_*i*_(^13^C, VANA-1) vs VPDB	*δ*_*i*_(^13^C, VANB-1) vs VPDB	*δ*_*i*_(^13^C, VANB-1) vs VANA-1 *	*δ*_*i*_(^13^C, VANB-1) vs VANA-1 *
	(700 MHz)	(700 MHz)	(700 MHz)	(400 MHz)
C1 C2 C3 C4 C5 C6 C7 C8	− 10.5 − 17.6 − 19.3 − 20.1 − 33.9 − 30.1 − 40.8 − 78.1	− 9.5 − 12.7 − 17.1 − 14.2 − 30.0 − 28.3 − 37.9 − 57.1	0.9 4.9 2.2 6.1 4.1 1.9 3.0 22.7	1.3 2.3 − 0.3 3.9 6.1 3.1 5.0 23.9

*Difference between the certified values. Note that *δ*_VANA_(VANB) = [1 + *δ*_VPDB_(VANB)]/[1 + *δ*_VPDB_(VANA)] − 1

### Further validation of the ^13^C results: gravimetric sample mixing

**Fig. 5:**
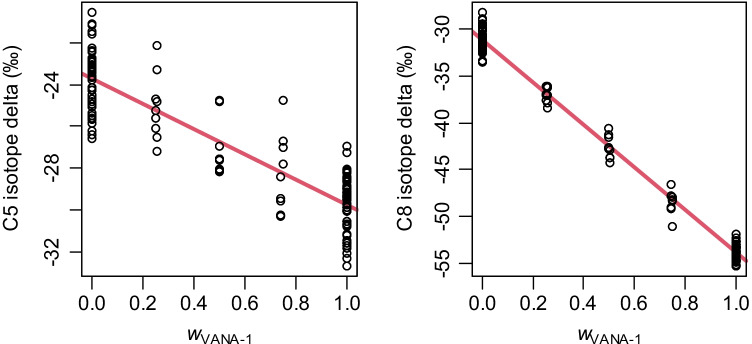
Isotope delta values for vanillin carbon atoms C5 and C8 measured from gravimetric mixtures of VANA-1 and VANB-1 at NRC. Horizontal axis shows the mass fraction of VANA-1 in such mixtures, *w*_VANA-1_ = *m*_VANA-1_/(*m*_VANA-1_ + *m*_VANB-1_)

At the NRC, we have performed additional analyses of VANA-1 and VANB-1 mixtures. In essence, the gravimetric mixing of the two materials should produce isotopic compositions that can be predicted beforehand from the mixing ratios and the isotopic composition of the two materials. Figure 5 shows the results for two carbon atoms (C5 and C8) whose results show a median residual of 0.85‰ from the linear model fits.

### Measurement uncertainty

We have evaluated the uncertainty associated with the site-specific carbon isotope values using several approaches. First, we have assessed the effect of the measurement model (Table 2), and the laboratory effect (Tables 5–6). Both show uncertainties below 1‰. Thus, we performed a Bayesian evaluation of the overall measurement uncertainty which incorporated uncertainty contributions from the isotopic composition of the VPDB, the reliability of peak area measurements (set to *u* = 2‰ as assessed from the experiments using doubly-labelled acetic acid), and the uncertainty associated with the bulk isotope value from the IRMS measurements (see Electronic Supplementary Material section S3). The analysis is shown in the Electronic Supplementary Material and leads to the measurement combined standard uncertainty of 0.7‰ (or expanded uncertainty of 1.4‰ at a 95% confidence level) for the individual carbon isotope values in either of the two vanillin materials.

### Homogeneity and stability of the materials

The batch homogeneity of carbon isotope values in VANA-1 and VANB-1 was assessed using the NRC measurements (Tables 3–4) and the aforementioned estimate of the measurement uncertainty (0.7‰) by fitting the random effects model to these data using *NIST Consensus Builder* [33]. The median uncertainty due to homogeneity, averaged over all carbon atoms and both materials, is *u*_hom_ = 0.3‰.

An isochronous short-term stability of VANA-1 and VANB-1 was conducted to examine the effect of sample storage. For each material, two vials were stored, one in the oven (+ 40 °C) and one at room temperature. After two and four weeks, the bulk *δ*(^13^C) and carbon site-specific isotope delta values of each vial were measured at the NRC by IRMS and ^13^C-qNMR. No significant effect in the δ(^13^C) values was observed for the samples stored at + 40 °C.

### Summary results

Our evaluation of the uncertainties associated with the site-specific carbon isotope values for all carbon atoms in the two materials can be summarized as follows. The uncertainty due to characterization, *u*_char_ = 0.3‰, the uncertainty due to homogeneity, *u*_hom_ = 0.3‰, the uncertainty due to short-term stability, *u*_stab_ = 0.0‰, and the uncertainty due to laboratory effect, *u*_lab_ = 0.6‰ (1.0‰/√3). Combining these sources of uncertainty yields combined standard uncertainty *u* = 0.7‰ (*k* = 1) or expanded uncertainty *U* = 1.4‰ (*k* = 2) at the 95% confidence level, applicable to all carbon isotope delta values for both materials. Table 8 summarizes the results for both materials with the average NRC results from Tables 3–4.

**Table 8:** Summary of site-specific carbon isotope delta values of vanillin in VANA-1 and VANB-1 (relative to the VPDB) and their expanded uncertainties at 95% confidence

Carbon atom	VANA-1	VANB-1
C1	− 21.4 ± 1.4‰	− 20.1 ± 1.4‰
C2	− 32.1 ± 1.4‰	− 29.9 ± 1.4‰
C3	− 32.5 ± 1.4‰	− 32.8 ± 1.4‰
C4	− 28.3 ± 1.4‰	− 24.5 ± 1.4‰
C5	− 29.8 ± 1.4‰	− 23.9 ± 1.4‰
C6	− 29.4 ± 1.4‰	− 26.4 ± 1.4‰
C7	− 23.7 ± 1.4‰	− 18.8 ± 1.4‰
C8	− 53.2 ± 1.4‰	− 30.6 ± 1.4‰
IRMS average	− 31.30 ± 0.06‰	− 25.85 ± 0.05‰

## Conclusion

The combination of ^13^C-qNMR and IRMS can provide site-specific isotope values for each carbon atom in vanillin. Our measurements for VANA-1 and VANB-1 reference materials provide results that are reliable to within approx. 1‰. While such a precision is an order of magnitude larger than typical IRMS measurements can provide, it is sufficient to show significant differences between the isotopic compositions of the various vanillin carbon atoms in these materials, even showing significant differences within the benzene ring. Our certified carbon isotope values of VANA-1 and VANB-1 were obtained with a qualified Bruker 400 MHz NMR instrument. However, since these values may not be recovered when samples are measured on other NMR spectrometers, one can use VANA-1 as the delta-zero material for intramolecular carbon isotope measurements. To our knowledge, these measurements and the associated reference materials represent the current state of the art in the field and we believe that these two vanillin CRMs, both available from the NRC, will prove useful to the stable isotope community and contribute to detecting counterfeiting of vanillin and other materials of significant economic value.

## Supplementary Information

Below is the link to the electronic supplementary material.

Supplementary file1 (DOCX 72.9 KB)

Supplementary file2 (XLSX 181 KB)
