# Supplementary material for: Site-specific carbon isotope measurements of vanillin reference materials by nuclear magnetic resonance spectrometry
Source: Anal Bioanal Chem. 2022 Sep 13;414(24):7153–65. doi: 10.1007/s00216-022-04292-0 (PMC9482901; doi:10.1007/s00216-022-04292-0)
Supplement: Supplementary file 1 — Supplementary file1 (DOCX 72.9 KB) [file 216_2022_4292_MOESM1_ESM.docx]

**Supplement Information**

**Site-Specific Carbon Isotope Measurements of Vanillin Reference Materials by Nuclear Magnetic Resonance Spectrometry**

Phuong Mai Le^a^*, Estelle Martineau^b,c^, Serge Akoka^b^, Gerald Remaud^b^, Michelle M.G. Chartrand^a^, Juris Meija^a^*, and Zoltán Mester^a^.

*a Metrology, National Research Council Canada, 1200 Montreal road, Ottawa, ON, K1A 0R6, Canada*

*b Nantes Université, CNRS, CEISAM, UMR6230, F-44000 Nantes, France*

*c CAPACITÉS SAS, Nantes, France*

*Corresponding Authors: [PhuongMai.Le@nrc-cnrc.gc.ca](mailto:PhuongMai.Le@nrc-cnrc.gc.ca), Juris.Meija@nrc-cnrc.gc.ca

**1. Chemical purity of VANA-1 and VANB-1 by Quantitative ^1^H NMR**


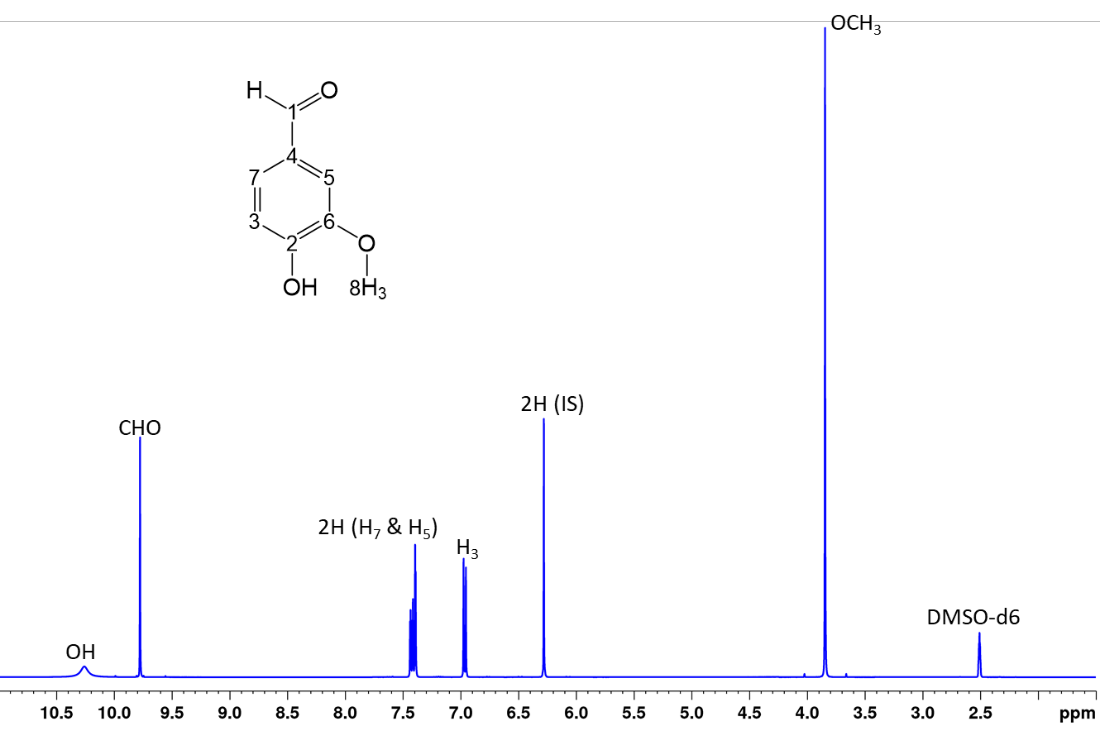


**Figure S1**: ^1^H NMR spectrum of Vanillin with maleic acid added as internal standard (IS)

The chemical purity of vanillin in VANA-1 and VANB-1 materials, $w_{\mathrm{vanillin}}$ was determined based on Eqs S1 and S2.

$w_{\mathrm{vanillin}}= \frac{n_{\mathrm{vanillin}\left( \mathrm{qNMR} \right)} \times M_{\mathrm{vanillin}}}{m_{\mathrm{vanillin}}}$ (S1)

$n_{vanillin(qNMR)}= \frac{n_{std(grav)}}{V_{\mathrm{std}}}\times\frac{I_{\mathrm{vanillin}}}{I_{\mathrm{std}}}\times\frac{p_{\mathrm{std}}}{p_{\mathrm{vanillin}}}\times v_{\mathrm{vanillin}}$ (S2)

*w*_vanillin_ mass fraction (purity) of VANA-1 or VANB-1

*n*_vanillin (qNMR)_ amount of VANA-1 or VANB-1 in the sample as determined by qNMR

*M*_vanillin_ molar mass of vanillin (*M*_vanillin_ = 152.1470 g/mol)

*m*_vanillin_ mass of VANA-1 or VANB-1

*n*_std(grav)_ amount of internal standard based on gravimetric data and known purity

*V*_std_ volume of internal standard solution

*I*_X_ integrated ^1^H peak area (X = vanillin or internal standard)

*p*_X_ number of protons corresponding to the integrated peak (X = vanillin or internal standard)

*V*_vanillin_ volume of vanillin solution

**Table S1**: ^1^H-qNMR results of three independent samples of VANA-1 on Bruker 400 MHz, using maleic acid as internal standard (3 replicate measurements per sample)

| Sample | 2H at 7.70 – 7.10 ppm | | 1H at 7.07 – 6.70 ppm | | 3H at 4.10 – | 3.60 ppm |
| --- | --- | --- | --- | --- | --- | --- |
|  | Mean (g/g) | *u*_2H_ (g/g) | Mean (g/g) | *u*_1H_ (g/g) | Mean (g/g) | *u*_3H_ (g/g) |
| VANA-spl1  VANA-spl2  VANA-spl3 | 0.9988  0.9984  0.9984 | 0.0001  0.0001  0.0003 | 0.9974  0.9978  0.9972 | 0.0008  0.0004  0.0006 | 0.9988  0.9985  0.9982 | 0.0001  0.0002  0.0004 |
| Average | 0.9985 | 0.0002 | 0.9974 | 0.0006 | 0.9985 | 0.0002 |

Arithmetic Mean: 0.9982 g/g with an uncertainty ± 0.0045 g/g

| Sample | 2H at 7.70 – 7.10 ppm | | 1H at 7.07 – 6.70 ppm | | 3H at 4.10 - | 3.60 ppm |
| --- | --- | --- | --- | --- | --- | --- |
|  | Mean (g/g) | *u*_2H_ (g/g) | Mean (g/g) | *u*_1H_ (g/g) | Mean (g/g) | *u*_3H_ (g/g) |
| VANB-spl1  VANB-spl2  VANB-spl3 | 0.9976  0.9972  0.9970 | 0.0006  0.0002  0.0004 | 0.9975  0.9977  0.9971 | 0.0006  0.0008  0.0007 | 0.9975  0.9970  0.9973 | 0.0005  0.0001  0.0002 |
| Average | 0.9973 | 0.0036 | 0.9974 | 0.0007 | 0.9973 | 0.0003 |

Arithmetic Mean: 0.9973 g/g with an uncertainty ± 0.0045 g/g,

**2. Correction factor for the calibration of carbon isotope composition obtained on 700 MHz to 400 MHz spectrometer using VANA-1 as reference values**

The correction factor for each carbon of vanillin as follows:

(i) Calculation of average value (*a*) of reduced molar fraction (*f_i_*/*F*_i_) for each carbon of VANA-1 samples measured on 400 MHz spectrometer;

(ii) Calculation of average value (*b*) of reduced molar fraction (*f_i_*/*F*_i_) for each carbon of all VANA-1 samples measured on the 700 MHz spectrometer.

(iii) The correction factor *k_cf_* for the homogenization of the results (obtained in Ottawa versus Nantes) towards the absolute *δ_i_*(^13^C) values is *(k_cf_)* = *a*/*b*

This *k_cf_* was applied to correct specific isotopic carbon values of VANB-1 and VANA-1 measured on 700 MHz spectrometer.

**Table S2**: Measurements of VANA-1 and VANB-1 on 700 MHz NMR instrument (5 samples and 5 replicate measurements per sample)

|  | 700 MHz vs VPDB | | 700 MHz vs VANA-1 | | Certified values* | |
| --- | --- | --- | --- | --- | --- | --- |
| Carbon atom | *δ_i_*(^13^C, VANA-1) | *δ_i_*(^13^C, VANB-1) | *δ_i_*(^13^C, VANA-1) | *δ_i_*(^13^C, VANB-1) | *δ_i_*(^13^C, VANA-1) | *δ_i_*(^13^C, VANB-1) |
| C1  C2  C3  C4  C5  C6  C7  C8 | -10.47  -17.56  -19.30  -20.10  -33.94  -30.10  -40.78  -78.10 | -9.53  -12.73  -17.10  -14.16  -29.98  -28.28  -37.87  -57.14 | -21.56  -31.96  -32.45  -28.05  -29.64  -29.25  -23.60  -53.87 | -20.64  -27.20  -30.28  -22.15  -25.66  -27.44  -20.63  -32.34 | **-21.36**  **-32.09**  **-32.52**  **-28.26**  **-29.84**  **-29.38**  **-23.73**  **-53.25** | **-20.10**  **-29.87**  **-32.77**  **-24.48**  **-23.85**  **-26.38**  **-18.82**  **-30.59** |

**3. Bayesian evaluation of the measurement uncertainty for carbon isotope delta values in VANA-1 sample from vial 170 using Stan programming language in R**

### DATA

N = 5; delta_g = -31.3; u_delta_g = 0.03; bias = 0.002;

R_VPDB = 0.011108; u_R_VPDB = 0.000010

area = (5x8 matrix)

718428139 703580573 701635468 697506173 705527019 704073454 708383604 701363128

717512556 703220069 702349410 697569369 703881649 705920174 710940931 701792179

720586656 704503309 703713989 699851573 706533412 707090753 712974041 704761635

721022412 703375432 704815965 698895199 707138418 706331654 709777512 704194793

719602725 705209613 703670662 699460598 706598123 706648052 711512718 704013155

### STATISTICAL MODEL

mod='

data{

int<lower=1> N; // Number of replicate sample measurements

matrix[N,8] area; // Matrix of the 8 carbon peak areas for each replicate

real delta_g; // Bulk carbon isotope delta from IRMS

real<lower=0> u_delta_g; // Uncertainty of the bulk carbon isotope delta from IRMS

real R_VPDB; // 13C/12C isotope ratio of VPDB

real<lower=0> u_R_VPDB; // Uncertainty of 13C/12C isotope ratio of VPDB

real<lower=0> bias; // Average bias/uncertainty for each peak area measurement

}

parameters{

vector<lower=0, upper=1>[8] x13; // Carbon-13 abundance for each atom

real<lower=0> s0; // Overall NMR sensitivity

real<lower=0> u_s0; // Variations of the sensitivity between the sample measurements

vector<lower=0>[N] s; // NMR sensitivity factor for each replicate run

real<lower=0> R_VPDB_hat; // 13C/12C for VPDB with its uncertainty

}

transformed parameters{

vector[8] k;

vector[8] x12;

vector[8] ab1;

matrix[8,8] ab2;

matrix[N,8] area_mc;

real delta_i[8];

real delta_g_hat;

for(i in 1:8) x12[i] = 1 - x13[i];

for(i in 1:8) ab1[i] = x13[i] * prod(x12) / x12[i];

for(i in 1:8) for(j in 1:8) ab2[i,j] = x13[i] * x13[j] * prod(x12) / (x12[i] * x12[j]);

k[1] = ab1[1] + ab2[1,2] + ab2[1,3] + ab2[1,5] + ab2[1,6] + ab2[1,7] + ab2[1,8];

k[2] = ab1[2] + ab2[2,1] + ab2[2,4] + ab2[2,5] + ab2[2,7] + ab2[2,8];

k[3] = ab1[3] + ab2[3,1] + ab2[3,4] + ab2[3,5] + ab2[3,6] + ab2[3,8];

k[4] = ab1[4] + ab2[4,2] + ab2[4,3] + ab2[4,6] + ab2[4,8];

k[5] = ab1[5] + ab2[5,1] + ab2[5,2] + ab2[5,3] + ab2[5,7] + ab2[5,8];

k[6] = ab1[6] + ab2[6,1] + ab2[6,3] + ab2[6,4] + ab2[6,7] + ab2[6,8];

k[7] = ab1[7] + ab2[7,1] + ab2[7,2] + ab2[7,5] + ab2[7,6] + ab2[7,8];

k[8] = ab1[8] + ab2[8,1] + ab2[8,2] + ab2[8,3] + ab2[8,4] + ab2[8,5] + ab2[8,6] + ab2[8,7];

for(i in 1:8) delta_i[i] = 1000 * ((x13[i]/x12[i])/R_VPDB_hat - 1);

delta_g_hat = mean(delta_i);

for(i in 1:N) for(j in 1:8) area_mc[i,j] = 1e10 * s[i] * k[j];

}

model{

x13 ~ normal(0.0107, 0.0003); // Weak prior

s0 ~ normal(7, 10); // Weak prior

u_s0 ~ cauchy(0, 10); // Weak prior

R_VPDB ~ normal(R_VPDB_hat, u_R_VPDB);

delta_g ~ normal(delta_g_hat, u_delta_g);

s ~ normal(s0, u_s0);

for(i in 1:N) for(j in 1:8) area[i,j] ~ normal(area_mc[i,j], bias * area[i,j]);

}

'

### INFERENCE

require(rstan)

df = list(N=nrow(area), area=area, delta_g = -31.30, u_delta_g = 0.06/2, bias = 0.002, R_VPDB = 11108e-6, u_R_VPDB = 10e-6)

init = function() list(area_mc = area, x13 = rep(0.0107, 8), s0 = 6.74, u_s0 = 0.01, s = rep(6.74, df$N), R_VPDB_hat = 11108e-6)

fit=stan(model_code = mod, data=df, init = init, iter=10000, cores=4, chains=4)

print(fit, pars=c('delta_i'), digits=4)

### RESULTS

Inference for Stan model: 8933899f13525f50d0b266462c7e0452.

4 chains, each with iter=10000; warmup=5000; thin=1;

post-warmup draws per chain=5000, total post-warmup draws=20000.

mean se_mean sd 2.5% 25% 50% 75% 97.5% n_eff Rhat

delta_i[1] -20.8757 0.0052 0.8236 -22.5048 -21.4263 -20.8734 -20.3202 -19.2399 25526 0.9999

delta_i[2] -32.2511 0.0050 0.8210 -33.8708 -32.8024 -32.2569 -31.7022 -30.6228 26839 1.0000

delta_i[3] -33.1980 0.0051 0.8098 -34.7874 -33.7492 -33.1990 -32.6469 -31.6123 25347 1.0000

delta_i[4] -29.4310 0.0052 0.8084 -31.0078 -29.9756 -29.4281 -28.8903 -27.8532 24436 0.9999

delta_i[5] -29.4998 0.0050 0.8059 -31.0788 -30.0377 -29.4989 -28.9594 -27.9097 26082 0.9999

delta_i[6] -29.4176 0.0051 0.8147 -31.0071 -29.9683 -29.4202 -28.8622 -27.8252 25087 0.9999

delta_i[7] -22.9072 0.0053 0.8261 -24.5192 -23.4657 -22.9072 -22.3516 -21.2932 24257 1.0000

delta_i[8] -52.8191 0.0050 0.8120 -54.4096 -53.3677 -52.8220 -52.2686 -51.2201 25855 0.9999

Samples were drawn using NUTS(diag_e) at Mon Jul 04 11:40:48 2022.

For each parameter, n_eff is a crude measure of effective sample size, and Rhat is the potential scale reduction factor on split chains (at convergence, Rhat=1).
